# Stoichiometric analysis of competing intermolecular hydrogen bonds using infrared spectroscopy†

**DOI:** 10.1039/c8ra02919a

**Published:** 2018-06-27

**Authors:** Ian Seungwan Ryu, Xiaohui Liu, Ying Jin, Jirun Sun, Young Jong Lee

**Affiliations:** Biosystems and Biomaterials Division, National Institute of Standards and Technology Gaithersburg Maryland 20899 USA yjlee@nist.gov; Volpe Research Center, American Dental Association Foundation Gaithersburg Maryland 20899 USA jsun@nist.gov

## Abstract

We quantitatively analyze multiple hydrogen bonds in mixtures of two monomers: urethane dimethacrylate (UDMA) and triethylene glycol-divinylbenzyl ether (TEG-DVBE). The carbonyl stretching band in infrared (IR) absorption spectra is deconvoluted into free and hydrogen-bonded carbonyl groups. The amounts of the sub-components are determined for 21 mixture compositions and initially analyzed using a simple stoichiometric model (based on one dominant hydrogen acceptor group per monomer species) for the equilibrium state of hydrogen bond formation. However, our in-depth stoichiometric analysis suggests that at least two UDMA acceptor groups (carbonyl and alkoxy oxygens) and one TEG-DVBE acceptor group (ether oxygen) contribute to intermolecular hydrogen bonding interactions. This finding is further supported by a quantitative analysis of the hydrogen bonding effect on the N–H stretching band. Moreover, the equilibrium constants of these hydrogen bond formations confirm that the inter-association between UDMA and TEG-DVBE is non-negligible in comparison to the UDMA self-associations. Such quantitative information on intermolecular interactions provides insight into the effect of hydrogen bonding on the copolymerization kinetics of these monomer mixtures.

## Abstract 2

Stoichiometric analysis of infrared spectra from UDMA and TEG-DVBE mixtures provides quantitative information on competing hydrogen bonds and intermolecular interactions in equilibrium.

## Graphical abstract

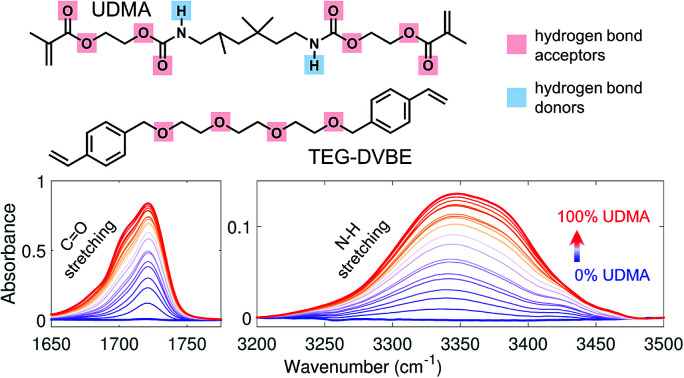

## Introduction

Hydrogen bonding strongly affects the chemical and physical properties of a wide range of materials, from small molecules, such as water and alcohol, to complex macromolecules, such as proteins and polymers.^1–4^ Accurate identification and quantitative characterization of hydrogen bonds are vital in understanding and controlling the material properties for intended mechanical, chemical, and biological applications. In a complex molecular system, such as polymers, characterizing the hydrogen bond effect is more challenging because of the inherent heterogeneity in the molecular interaction of the material and the specific and directional nature of hydrogen bonding. As a quantitative and non-invasive tool, vibrational spectroscopic methods, including IR and Raman spectroscopy, have been widely used to study hydrogen bonding.^5,6^ Peak position shift and intensity change observed by vibrational spectroscopy indicate the strength and the amount of the hydrogen bonds associated with the specific hydrogen donors and acceptors because formation of a hydrogen bond makes the vibrational frequencies red-shifted for both the hydrogen donors and the hydrogen acceptors, and a stronger bond tends to shift the frequency further to the red.^7,8^ Previously, Painter, Coleman, and their co-workers used IR spectroscopy to describe the equilibrium of hydrogen bond formations in urethane–ether polymer blends.^9–11^ Based on the IR results, they successfully explained the thermodynamics of inter-association and self-association using a stoichiometric model.

New development in polymer chemistry demands a better understanding of the intermolecular interactions of monomers and their impact on polymerization reaction and resulting polymer networks. Recently, Sun *et al.* reported a rapid, composition-controlled photo-copolymerization of a methacrylate-based monomer, urethane dimethacrylate (UDMA), and a styrene-based monomer, triethylene glycol-divinylbenzyl ether (TEG-DVBE).^12–14^ Such well-controlled copolymerization overcomes the diffusion limitation, which is typically observed in copolymerizations of monomers with distinct viscosities.^15–18^ In general, the low viscosity monomer tends to polymerize greater at a high degree of conversion as a result of relatively faster diffusion in the increasingly condensed polymer network. In the UDMA/TEG-DVBE photo-copolymerization system, the feeding monomer composition was maintained up to 90% degree of conversion even though the viscosity of UDMA is approximately 240 times higher than that of TEG-DVBE. One can hypothesize that hydrogen bonding plays vital roles in this rapid, composition-controlled copolymerization *via* lowering the activation energy through hydrogen bonding to the carbonyl functional group of methacrylate^19^ or increasing the collision frequency in a preferred orientation, *e.g.*, *via* pre-association reinforced by hydrogen bonding.^20^

In this study, multiple intermolecular hydrogen bonding interactions are identified using peak fitting of the C=O and the N–H bands from IR spectra of UDMA/TEG-DVBE mixtures. We propose two stoichiometric models: one dominant acceptor group per monomer species, similar to the Painter–Coleman model; and multiple competing acceptor groups per monomer species. Changes in the free and hydrogen bonded carbonyl peak areas with respect to the molar ratio of UDMA and TEG-DVBE are analyzed by the two proposed stoichiometric models. We discuss the effect of the competing acceptors of UDMA on the UDMA self-association and the UDMA–TEG-DVBE inter-association in the context of their contribution to the reaction kinetics of the rapid composition-controlled copolymerization.

## Materials and methods

Triethylene glycol-divinylbenzyl ether (TEG-DVBE) was synthesized from triethylene glycol and 4-vinylbenzyl chloride and purified in-house.^12^ Urethane dimethacrylate (UDMA) was used as received from Esstech (Essington, PA, USA). Nineteen liquid mixtures of TEG-DVBE and UDMA were prepared at various compositions without any additional solvents added. The composition of each mixture was determined as a mole fraction by proton nuclear magnetic resonance (^1^H NMR) spectroscopy. The mole fractions were determined from the ratio of integrated areas of the TEG-DVBE vinyl protons at 6.71 ppm and the C=C protons at 6.13 ppm.

A Fourier transform infrared (FT-IR) spectrometer (Thermo, Nicolet Nexus 670) with an attenuated total reflection (ATR) adapter (PIKE Technology, GladiATR) was used to measure IR absorption spectra of the monomer mixtures at room temperature. A total of 128 scans were collected from 650 cm^−1^ to 4000 cm^−1^ with 4 cm^−1^ resolution. The artifact due to frequency-dependent ATR penetration depth was removed from the measured spectra by an advanced ATR correction algorithm provided by the spectrometer manufacturer. Prior to further peak analysis, each spectrum was detrended with a baseline calculated using the anchoring points at 1595 cm^−1^, 1785 cm^−1^, 3150 cm^−1^, and 3550 cm^−1^. The peak fitting and deconvolution were performed in the frequency range between 1650 cm^−1^ and 1780 cm^−1^ for the carbonyl band and between 3200 cm^−1^ and 3500 cm^−1^ for the amine band by the nonlinear curve fitting provided in OriginPro (OriginLab).

## Results and discussion

**Fig. 1:**
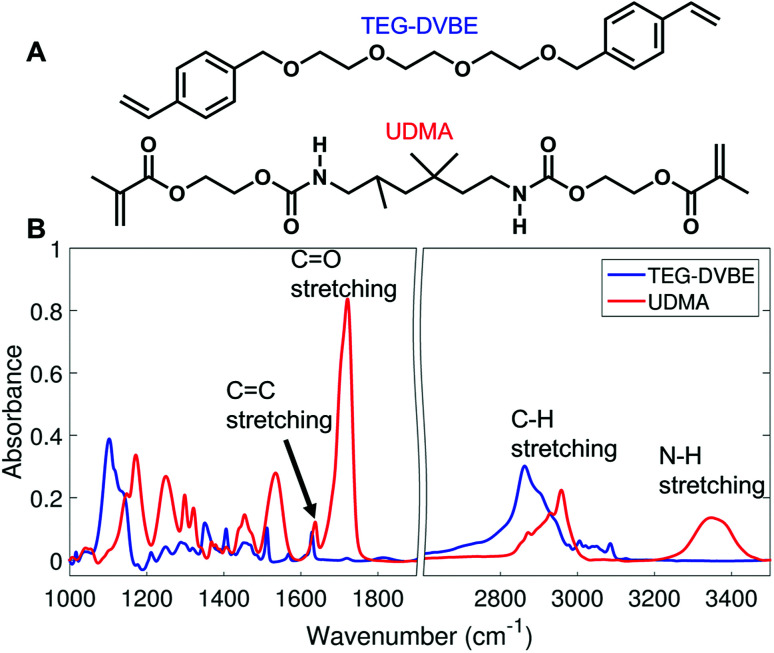
(A) Molecular structures of UDMA and TEG-DVBE. (B) Infrared (IR) spectra of the two studied monomers.

We monitored changes in the amount of hydrogen bonding by analyzing IR modes from different functional groups. From the molecular structures of UDMA and TEG-DVBE, shown in Fig. 1A, the N–H groups in UDMA are identified as a hydrogen donor group, while the oxygens in UDMA and TEG-DVBE are considered as hydrogen acceptor groups. From the IR spectra of the neat UDMA and TEG-DVBE monomers shown in Fig. 1B, two distinguishable UDMA peaks are well-separated from neighboring peaks. The first peak at 3350 cm^−1^ corresponds to the N–H stretching mode while the other peak at 1775 cm^−1^ corresponds to the C=O stretching mode.

**Fig. 2:**
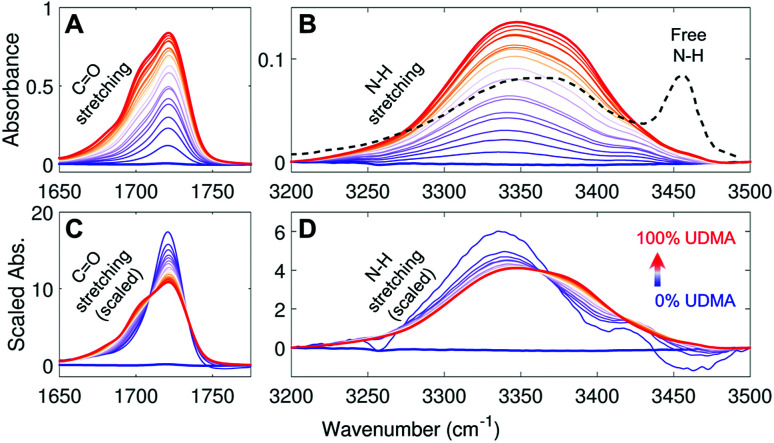
IR spectra of UDMA/TEG-DVBE mixtures with various compositions (A) in the C=O stretching region and (B) in the N–H stretching region. The black dashed line in (B) indicates the IR spectrum of free N–H measured from 0.2 M UDMA solution in CCl_4_ (digitized from Fig. 9 in ref. 21). (C) and (D) For better comparison, each spectrum in (A) and (B), respectively, is scaled by its peak area within the displayed spectral range. The UDMA mole fractions of the series of mixtures are measured by NMR as 0%, 3.9%, 9.1%, 14%, 19%, 20%, 29%, 31%, 42%, 48%, 53%, 58%, 63%, 67%, 73%, 77%, 83%, 87%, 90%, 95%, and 100%, from the blue curve to the red curve, respectively. The uncertainty of the mole fraction values is 1%, which determined by digitization precision for NMR area calculation.

Fig. 2A and B show IR spectra of the C=O and the N–H stretching modes measured from UDMA/TEG-DVBE mixtures with twenty-one different molar ratios. Because C=O and N–H bands both originate from UDMA, their absorbance increases monotonically with the UDMA mole fraction. The observed N–H absorption spectra in Fig. 2B show a broad peak located at 3350 cm^−1^ for all mixtures. We compared these spectra with a spectrum of a “free” (non-hydrogen-bonded) N–H. The black dashed line in Fig. 2B is digitized from an IR spectrum of a low concentration UDMA solution in CCl_4_, where the narrow peak at 3455 cm^−1^ is from the N–H in the state free from hydrogen bonding.^21^ The absence of the free N–H peak means that most of the N–H groups in the mixtures are in hydrogen bonded states. The dominant population of hydrogen bonded states of the N–H group can be explained by the excess of hydrogen bond acceptors in all mixture compositions. For example, two donors (N–H) from one UDMA molecule will encounter eight acceptors (four carbonyl oxygens and four alkoxy oxygens) from the same UDMA and additional acceptors from TEG-DVBE. The area-scaled spectra in Fig. 2D shows a shift in hydrogen bonding character of the N–H more clearly than that in Fig. 2B. As the mole fraction of UDMA increases, the contribution of the 3380 cm^−1^ peak increases while the contribution of the 3340 cm^−1^ peak decreases. This peak shift suggests that the 3380 cm^−1^ subcomponent corresponds to hydrogen bonding with other UDMA (self-association), while the 3340 cm^−1^ subcomponent corresponds to hydrogen bonding with TEG-DVBE (inter-association). More detailed N–H peak analysis is discussed with stoichiometric models later.

The IR spectra of the carbonyl (C=O) group provide a different metric of hydrogen bonding interactions. Because the total number of available acceptors is greater than that of the donors (N–H groups), not all carbonyl oxygens can form hydrogen bonds. Also, the carbonyl oxygens may compete with other acceptor groups from UDMA and TEG-DVBE depending on mixture composition. Even if all N–H groups happen to form hydrogen bonds exclusively with the carbonyl oxygen, still only half of the C=O groups will become hydrogen bonded and the other half of the C=O groups will remain in the “free” (non-hydrogen bonded) state. Then, the fraction of the free C=O will become 0.5. On the other hand, in the presence of excess TEG-DVBE, the fraction of the free C=O will increase toward one. The dominant presence of the free C=O can be found in the blue shifted C=O peak in the spectrum of the mixture of 3.9% UDMA in Fig. 2A. Change in the relative contribution of the free C=O can be better distinguished in the area-scaled spectra shown in Fig. 2C. As the mole fraction of UDMA increases, the contribution of the free C=O peak at 1720 cm^−1^ decreases, while that of the hydrogen bonded C=O peak below 1710 cm^−1^ increases. While both absorption peaks of C=O and N–H can be used for quantitative characterization of hydrogen bonds in the mixtures, we found that the C=O absorption spectra provide higher signal-to-noise ratios and have been better characterized than the N–H absorption band. Therefore, we analyzed the C=O band first to construct stoichiometric association models and later analyzed the N–H band to further validate the models.

### (a) Peak fitting of the C=O stretching band

**Fig. 3:**
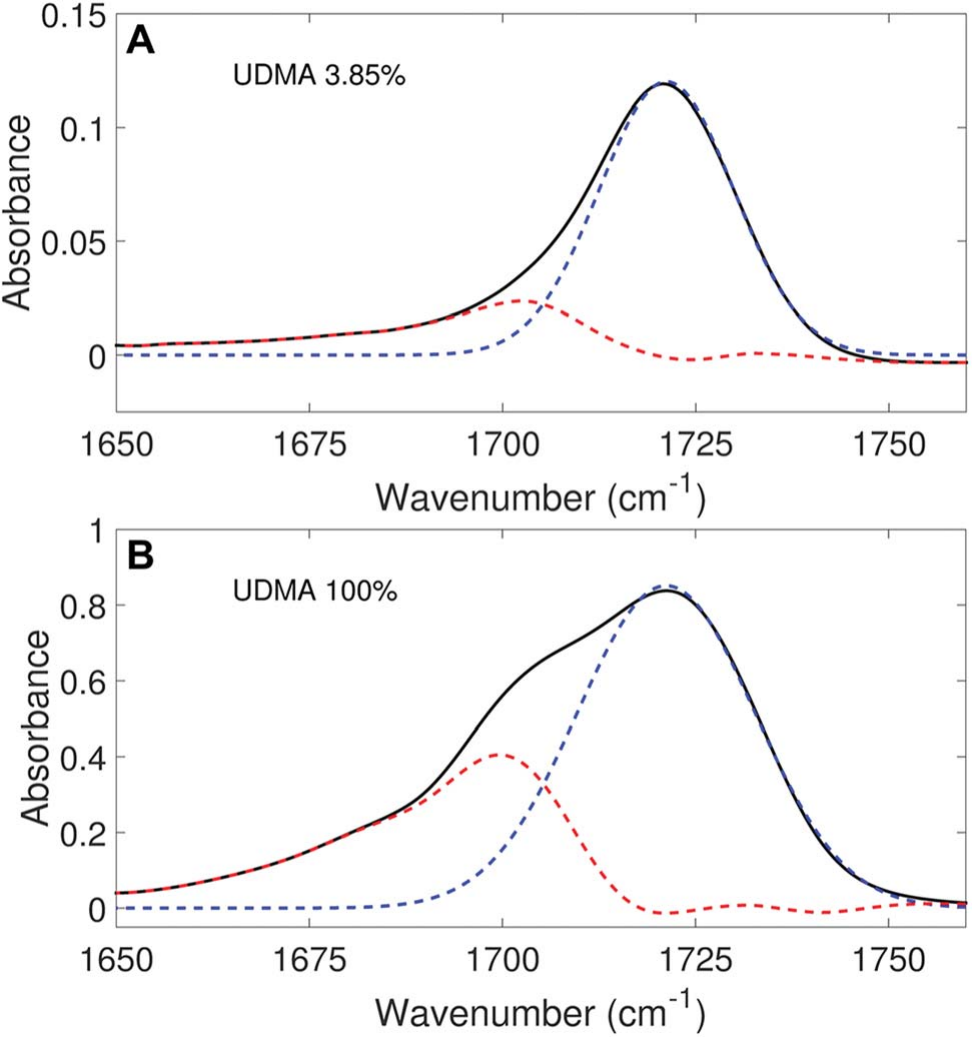
Peak fitting analysis of IR spectra in the carbonyl region. The black lines represent the experimental data, and the blue dashed lines represent the free C=O (1721 cm^−1^) band determined from the fitting analysis. The peak area (noted by the red dashed lines) outside of the free C=O band is considered as the hydrogen bonded C=O.

We aimed to deconvolute all twenty C=O spectra in Fig. 2A into common analytical functions for subcomponent peaks to quantify the free and hydrogen-bonded states. However, after we attempted multiple fitting approaches using various analytical functions (Lorentzian, Gaussian, and Voigt), we found it impractical to fit the measured spectra by the sum of a reasonable number (<4) of analytical functions with shared parameters (the center frequency and the full-width-half-maximum, FWHM). Alternatively, we employed a simple, semi-analytical deconvolution method. First, the free C=O state peak was calculated with a single Gaussian function with the center frequency and the FWHM determined from the spectrum of the lowest, non-zero UDMA mole fraction. As shown in Fig. 3A, the frequency region above 1715 cm^−1^ of the C=O absorption band is reasonably well fit with a single Gaussian function with the center frequency of 1721 cm^−1^ and the FWHM of 22 cm^−1^. The peak position and the width are consistent with the previously reported IR band of the free C=O state.^7,21,22^ For the rest of the spectra, the amplitude of the Gaussian function was determined by fitting only in the region between 1715 cm^−1^ and 1780 cm^−1^, where the free C=O contribution is dominant. After the free C=O component was determined from the Gaussian function, the hydrogen bonded C=O component was calculated by subtracting the Gaussian function from the observed spectrum. Fig. 3 shows examples of this semi-analytical binary deconvolution. The hydrogen bonded C=O subcomponent is not a symmetrical function, but its area represents the amount of the hydrogen bonded C=O.

The area of a subcomponent can be converted into the concentration when divided by its absorption coefficient. It was reported that a carbonyl group exhibits a higher absorption coefficient in the hydrogen bonded state than in the free state.^10,11^ To convert the area ratios into the concentration ratios of the free C=O to the hydrogen bonded C=O, we define the absorption coefficient ratio, *r* ≡ *ε*(C=O_HB_)/*ε*(C=O_free_), where *ε*(C=O_HB_) and *ε*(C=O_free_) denote the absorption coefficients of hydrogen bonded and free C=O, respectively. Then, from the Beer's law, the fraction of the free carbonyl group, *Φ*, can be expressed as follows.1
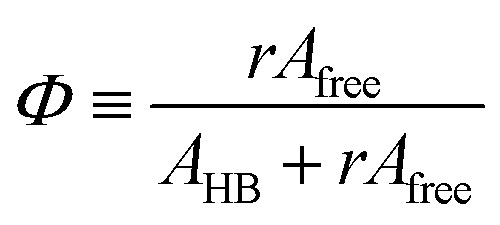


**Fig. 4:**
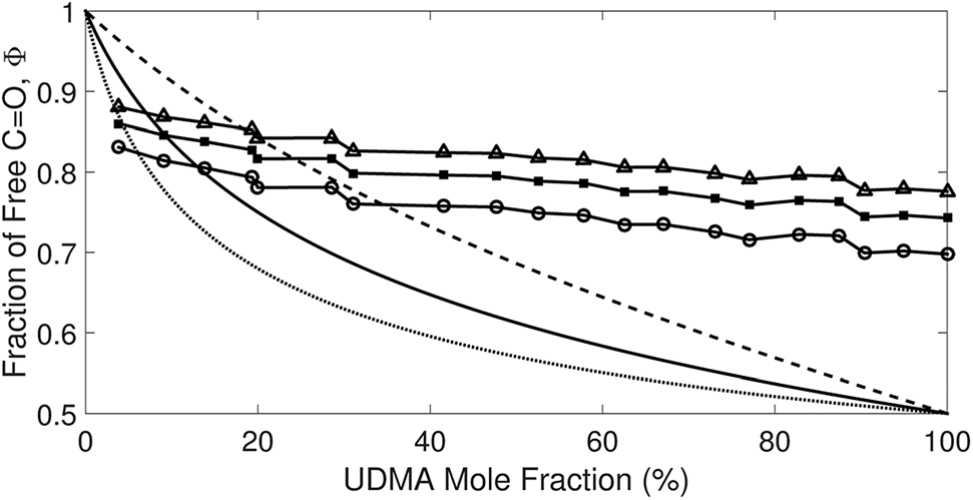
The fraction of the free C=O state determined by semi-analytical peak fitting of C=O spectra of various mixtures. Three different values of absorption coefficient ratio (*r*) are used to convert the subcomponent areas into *Φ*: *r* = 1.8 (triangle), 1.5 (circle), and 1.2 (square). The experimental results are compared with simulation curves calculated by the (1 + 1) model [see eqn (4)] with different *P* = 2 (dashed line), 5 (solid line), and 10 (dotted line).

*A*_free_ and *A*_HB_ represent the areas of the free and the hydrogen bonded C=O, respectively. Although multiple values of *r* have been reported in IR spectroscopic studies of C=O in various hydrogen bonded systems,^11^ the *r* value for this specific system has not yet been studied. Therefore, we assumed three values from the full range of reported values of *r* to represent its uncertainty. Scatter plots in Fig. 4 shows the series of *Φ* values calculated with *r* = 1.2, 1.5, and 1.8 as a function of the UDMA mole fraction. For all three *r* values, *Φ* decreases monotonically as the UDMA mole fraction increases, reconfirming that more C=O groups become hydrogen bonded because of less competition from TEG-DVBE.

### (b) Stoichiometric models of hydrogen bond formations

**Fig. 5:**
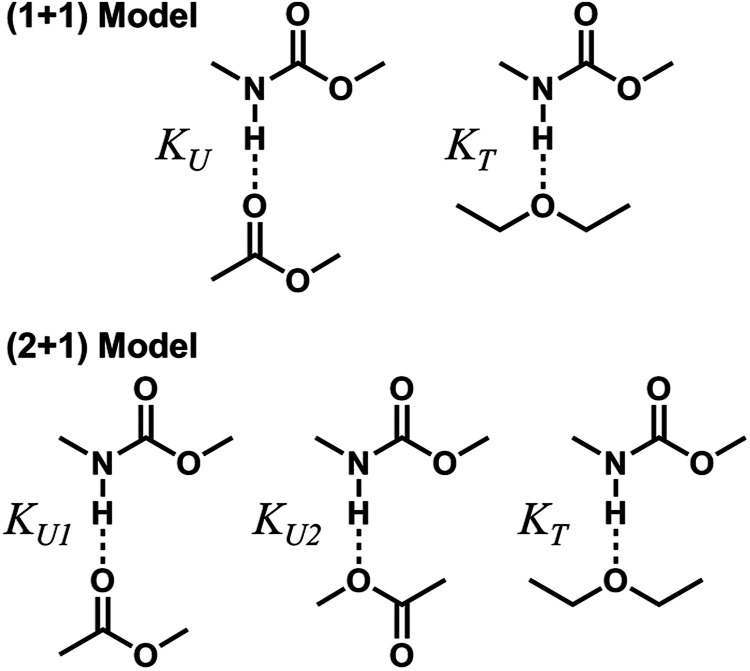
Hydrogen bond formation models in a mixture of UDMA and TEG-DVBE. The (1 + 1) model considers the UDMA carbonyl oxygens and the TEG-DVBE ether oxygens as available hydrogen acceptors. *K*_U_ denotes the equilibrium constant of hydrogen bond formation of a UDMA carbonyl oxygen and a UDMA amine hydrogen. *K*_T_ denotes the equilibrium constant of an ether oxygen of TEG-DVBE and a hydrogen of UDMA. The (2 + 1) model considers an additional hydrogen acceptor (the alkoxy oxygen) from UDMA in addition to the (1 + 1) model. *K*_U1_ and *K*_U2_ denote the hydrogen bonding equilibrium constants of the UDMA carbonyl and alkoxy oxygens, respectively.

To describe molecular association in the UDMA/TEG-DVBE mixtures, we considered two simplified stoichiometric models for hydrogen bonding equilibrium. These models assume that (1) all hydrogen donor groups and acceptor groups are independently accessible without geometric preference or restriction and that (2) the hydrogen bond formation equilibrium constants are identical for the same functional groups regardless of their locations within a molecule (Fig. 5). For example, the two ether oxygens closer to the styrene end and the other two closer to the center of TEG-DVBE molecule are counted as four independent, identical ether oxygens in the perspective of hydrogen bond formation with N–H groups. Likewise, one UDMA provides four independent, identical carbonyl oxygens and four independent, identical alkoxy oxygens. In addition, it should be noted that we did not consider the amine group nitrogen in UDMA as a hydrogen bond acceptor because its probability of hydrogen bond formation was estimated to be lower than 0.01%.^23^

#### (1 + 1) model – one type of UDMA acceptor and one type of TEG-DVBE acceptor

As discussed above in Fig. 2, at any composition of UDMA/TEG-DVBE, all N–H groups are in the hydrogen bonded state. The shifts and shape changes in N–H and C=O bands with varying UDMA mole fraction (Fig. 2) suggest that hydrogen bond acceptors in both UDMA and TEG-DVBE participate in hydrogen bonding interaction. In particular, the changes in the C=O band demonstrate a strong involvement of C=O groups in the molecular interactions in UDMA/TEG-DVBE mixtures. We considered the simplest model addressing these observations. The UDMA hydrogen donor (N–H) can form a bond with either one of two types of acceptors: one from the UDMA carbonyl oxygens and the other from the TEG-DVBE ether oxygens. This simplest (1 + 1) model considers one UDMA self-association and one UDMA⋯TEG-DVBE inter-association. Their equilibrium constants, *K*_U_ and *K*_T_, are defined as2
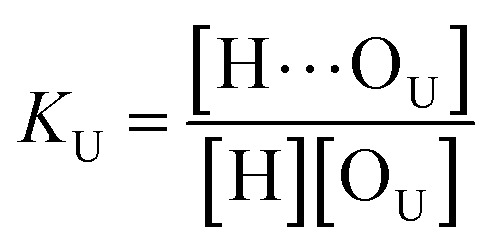
3
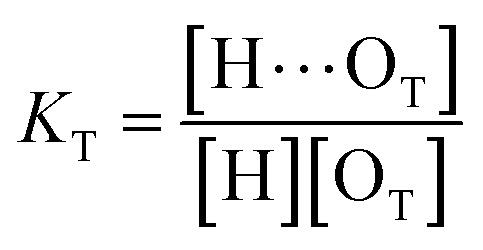
where [H], [H⋯O_U_], [O_U_], [H⋯O_T_], and [O_T_] represent the concentrations of the free N–H, the hydrogen bonded carbonyl, the free carbonyl, the hydrogen bonded ether, and the free ether, respectively. We use the ratio of these two equilibrium constants,4
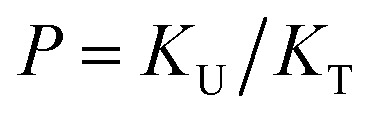
to quantify the relative reactivity between the two hydrogen bond formations.

Based on the above-mentioned assumptions of independence and equivalence of all hydrogen acceptor and donor groups, we can write the following relations:5
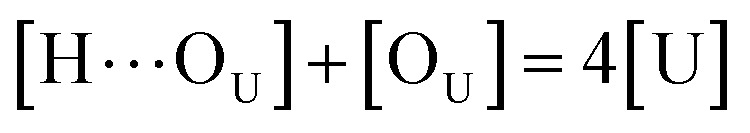
6
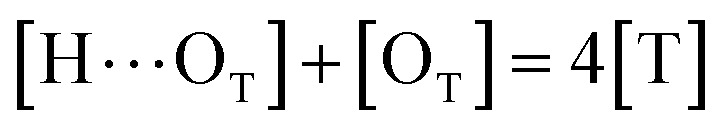
7
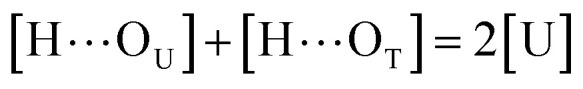
where [U] and [T] are the concentrations of UDMA and TEG-DVBE, respectively. The mole fraction of UDMA in a mixture is expressed as *ϕ*_U_ = [U]/([U]+[T]). Using eqn (2)–(7), the fraction of the free carbonyl group, *Φ*, can be expressed with only a single unknown parameter *P*. (see the ESI† for the derivation).8
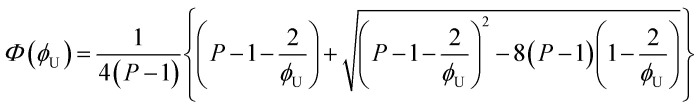


The curves in Fig. 4 show *Φ* calculated as a function of *ϕ*_U_ with three different *P* values. The *Φ* value decreases when *ϕ*_U_ increases because N–H is more likely surrounded by UDMA than by TEG-DVBE. The greater the value of *P*, the earlier the value of *Φ* reaches a plateau. However, none of these model curves reproduced the experimentally determined *Φ* values. It is noted that when *ϕ*_U_ approaches one, all model curves converge at 0.5, which is evidently smaller than the experimental values (0.7 < *Φ* < 0.8). This indicates that more than half of C=O exists in the free state even when all N–H forms hydrogen bonding. This is not possible when the C=O is assumed to be the sole acceptor in UDMA because it takes half of the all C=O groups (four per UDMA) to form hydrogen bonds with all N–H groups (two per UDMA) at *ϕ*_U_ = 1. In other words, the (1 + 1) model cannot explain the experimental results without considering an additional hydrogen bond acceptor from UDMA.

#### (2 + 1) model – two types of UDMA acceptors and one type of TEG-DVBE acceptor

As shown in Fig. 5, the alkoxy oxygen in UDMA can be considered as an additional hydrogen acceptor. Then, the equilibrium constants are defined differently for the UDMA hydrogen bond formation as follows:9
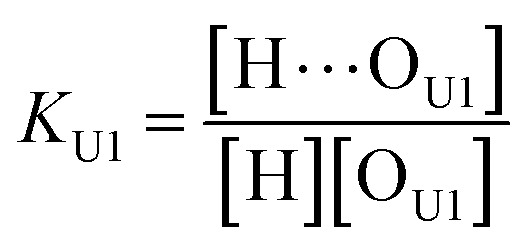
10
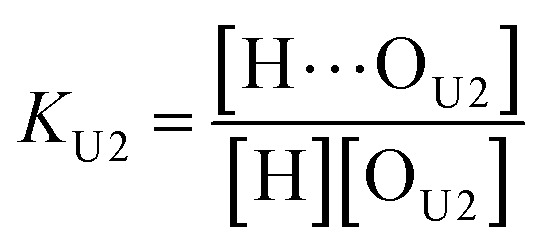


[O_U1_] and [O_U2_] are the concentrations of free carbonyl and free alkoxy oxygens in UDMA, respectively. [H⋯O_U1_] and [H⋯O_U2_] are the concentrations of the hydrogen-bonded carbonyl and hydrogen-bonded alkoxy oxygens in UDMA, respectively. These concentrations are related by the following stoichiometric relations:11
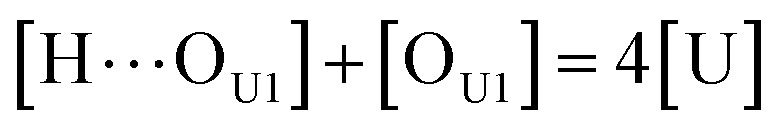
12
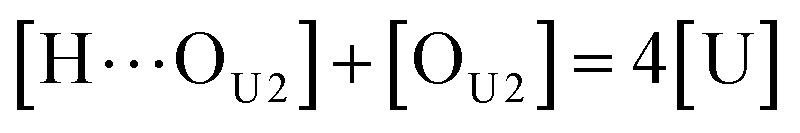
13
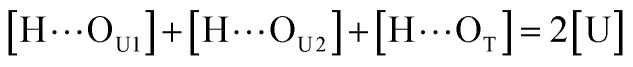


In the (2 + 1) model, we define two ratios with the three equilibrium constants:14
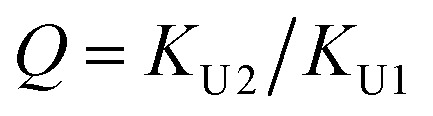
15
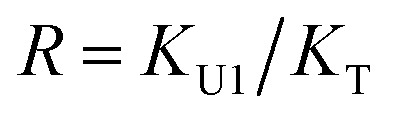


Due to the additional species, the (2 + 1) model cannot be analytically solved in the form of *Φ*(*ϕ*_U_). Instead, we obtained its inverse functional form *ϕ*_U_(*Φ*) as follows (see the ESI† for the derivation):16
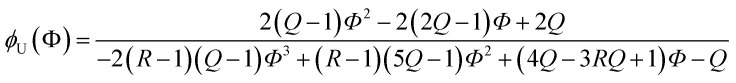


Out of the two ratios, *Q* and *R*, the self-association ratio *Q* can be determined from the experimental value *Φ* observed at *ϕ*_U_ = 1. When only UDMA is present, [T] = [H⋯*O*_T_] = [O_T_] = 0, and by eqn (13) and (14), the parameter *Q* can be expressed as17
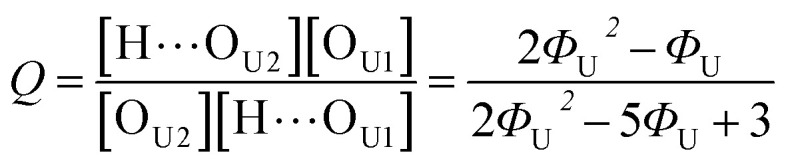
where *Φ*_U_ denotes the *Φ* value measured at *ϕ*_U_ = 1. Once the ratio *Q* is determined at *ϕ*_U_ = 1, we determined the ratio *R* by visually comparing a curve from eqn (16) with the experimental results for the best match.

**Fig. 6:**
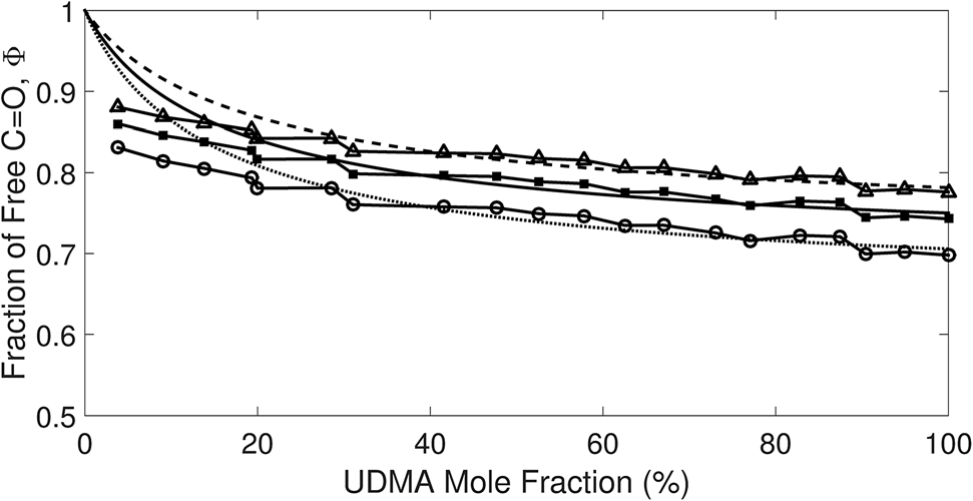
Comparison of the experimental results and the best fit curves of the (2 + 1) model by using eqn (16). The best fit parameters for the three series of data are: *Q* = 1.4 and *R* = 3 for *r* = 1.8; *Q* = 1.0 and *R* = 4 for *r* = 1.5; and *Q* = 0.62 and *R* = 5 for *r* = 1.2. The absorption coefficient ratio (*r*) in the fitting analysis was set to 1.8 (triangle), 1.5 (circle) and 1.2 (square).

The comparison shown in Fig. 6 returns *Q* = 1.0 ± 0.4 and *R* = 4 ± 1 for 1.2 < *r* < 1.8. First, the *Q* (= *K*_U2_/*K*_U1_) value of 1.0 ± 0.4 indicates that the two types of hydrogen bonds ([N–H⋯O_U1_] and [N–H⋯O_U2_]) are present in the similar amount. In a simulation study, the probability of hydrogen bonding to the alkoxy oxygen in urethane was calculated to be very low due to a specific molecular configuration.^23^ An X-ray study also found no evidence of hydrogen bonding to the alkoxy oxygen in a polyurethane crystal.^24^ In other IR studies, however, the strength of hydrogen bonding to the alkoxy oxygen was found to be comparable to the hydrogen bond strength to the carbonyl oxygen in a carbamate group.^25,26^ Our model, based on the C=O and N–H absorption bands, strongly suggests that hydrogen bonding of the alkoxy oxygen is comparable to that of the carbonyl oxygen in UDMA. Second, the ratio of the equilibrium constants of the self-and inter-association is represented by *R* (= *K*_U1_/*K*_T_). The *R* value of 4 ± 1 suggests that the hydrogen bonding to the ether oxygen in TEG-DVBE is a little less probable than those to the carbonyl and alkoxy oxygens in UDMA. However, the inter-association between UDMA⋯TEG-DVBE is not negligible compared to the self-association of UDMA as their equilibrium constants are comparable within one order of magnitude. This is consistent with previous reports on comparative studies of hydrogen bonding of carbonyl and ether groups.^8,9^ The determined values of *Q* and *R* are used to calculate the relative amounts of the hydrogen bond related species in a mixture. Table 1 shows quantitative snapshots of the functional groups involved in hydrogen bonding interaction in a mixture solution based on the (2 + 1) model.

**Table 1:** The relative amounts of hydrogen bond related species calculated for *Q* = 1 and *R* = 4 based on the (2 + 1) model at three different UDMA mole fractions

	*ϕ*_U_ = 0.25	*ϕ*_U_ = 0.5	*ϕ*_U_ = 0.75
	*Φ* = 0.83	*Φ* = 0.78	*Φ* = 0.76
O_U1_ (free carbonyl oxygen)	17%	26%	33%
O_U2_ (free alkoxy oxygen)	17%	26%	33%
O_T_ (free ether oxygen)	57%	31%	13%
H⋯O_U1_ (hydrogen bonded carbonyl oxygen)	3%	7%	10%
H⋯O_U2_ (hydrogen bonded alkoxy oxygen)	3%	7%	10%
H⋯O_T_ (hydrogen bonded ether oxygen)	3%	2%	1%

### (c) Peak fitting of the N–H stretching band

**Fig. 7:**
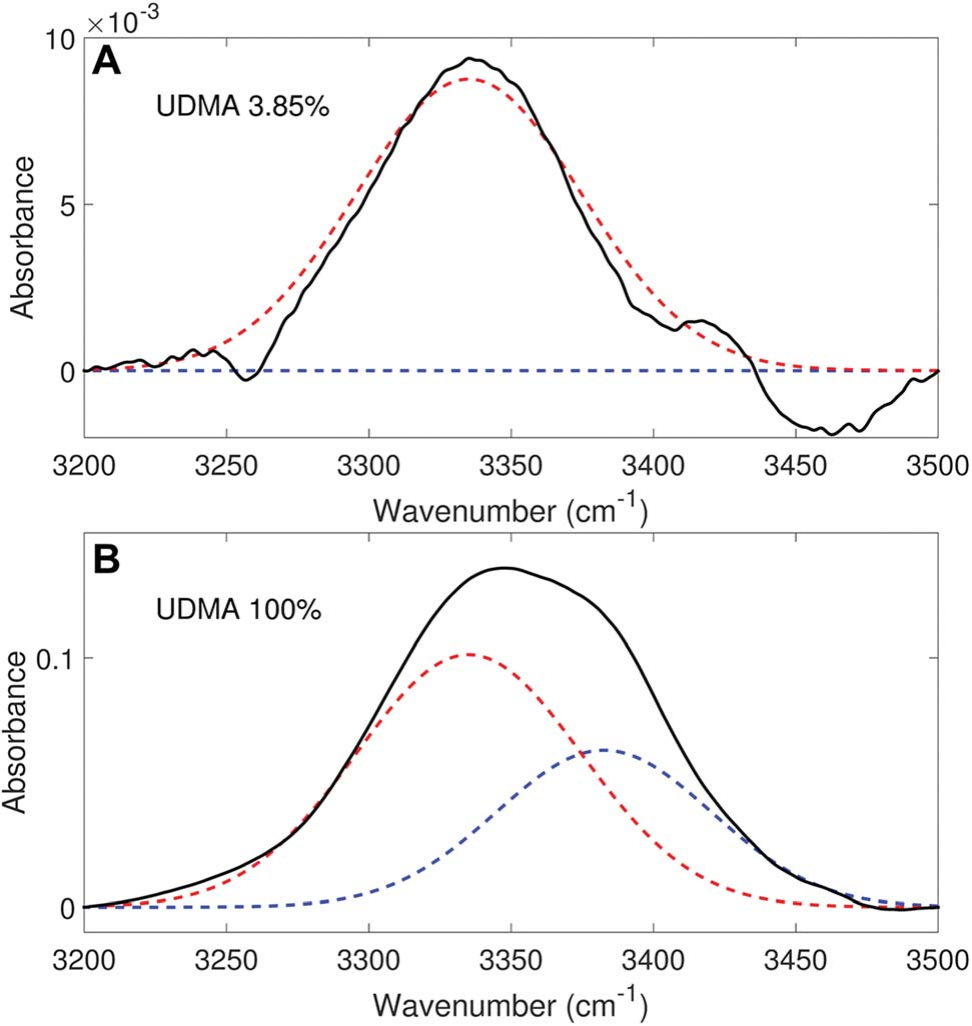
Peak fitting analysis of the amine (N–H) stretching band. The black lines represent the experimental data. The red dashed lines represent a Gaussian function centered at 3383 cm^−1^ with the FWHM of 75 cm^−1^, and the blue dashed lines, another Gaussian function centered at 3335 cm^−1^ with the FWHM of 79 cm^−1^.

**Fig. 8:**
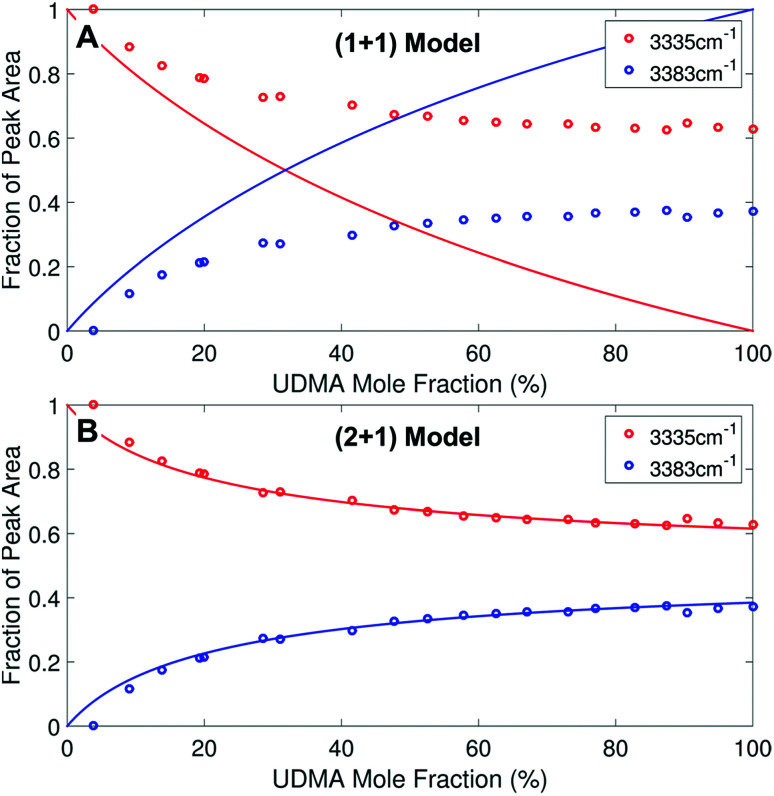
Area fractions (circles) of the two subcomponents in the N–H stretching band. (A) Calculated fractions based on the (1 + 1) model for *P* = 4. (B) Calculated fractions based on the (2 + 1) model for *Q* = 1 and *R* = 4. For the model calculations, the absorption coefficient is assumed to be 1.6 times larger for the 3335 cm^−1^ peak than for the 3383 cm^−1^ peak.

We analyzed the N–H stretching band in the region between 3200 cm^−1^ and 3500 cm^−1^ to validate the stoichiometric interpretations based on the C=O band. As discussed for Fig. 2B and D, all N–H groups are involved in hydrogen bonding, and their peak shape changes as the mixture composition varies. Interestingly, the observed N–H spectra are all reasonably well fit with two Gaussian functions with the center frequencies and the FWHMs shared for all mixture compositions. We find that fitting with more than two subcomponents returns unreliable results due to the broad bandwidth and the proximity of potential subcomponents. Fig. 7 shows two examples of the peak fitting performed at two very different UDMA mole fractions. At the lowest UDMA mole fraction in Fig. 7A, where most of the N–H groups form hydrogen bonding with the TEG-DVBE ether oxygens, the peak is fitted with a single Gaussian centered at 3335 cm^−1^. The subcomponent at 3335 cm^−1^ can be easily identified as the N–H group associated with TEG-DVBE. As UDMA mole fraction increases, the fraction of the 3335 cm^−1^ peak decreases, while the contribution from the other peak centered at a higher frequency of 3383 cm^−1^ increases. Therefore, the 3383 cm^−1^ peak can be identified as the N–H group associated with UDMA. This monotonic behavior of the two subcomponents may seem to be more consistent with the (1 + 1) model than the (2 + 1) model. However, the subcomponent fractions calculated with the (1 + 1) model showed a significant discrepancy from the observed ones (Fig. 8A). As the UDMA mole fraction increases, the discrepancy between the observed and calculated values becomes larger. The two subcomponents under the N–H band observed at the 100% UDMA mole fraction cannot be explained by the (1 + 1) model without considering multiple types of hydrogen bonding in the 100% UDMA sample.

The (2 + 1) model, which considers two types of hydrogen acceptors from UDMA and one type of hydrogen acceptor from TEG-DVBE, can explain the two peaks from the 100% UDMA spectrum as well as the one peak from the TEG-DVBE excess spectrum. The successful application of the two-peak global fitting for all N–H spectra supports the idea that the peak of the N–H bound to TEG-DVBE closely overlaps one of the two peaks bound to UDMA. In Fig. 8B, the fraction of each peak area was calculated based on the (2 + 1) model using the previously determined parameters, *Q* and *R*. For the calculation, the ratio of the absorption coefficients of the N–H subcomponents is needed. Similar to the C=O absorption band, hydrogen bonding changes the absorption coefficient as well as the vibrational frequency. Skrovanek *et al.* reported that from a temperature dependent IR study of polyamide, the absorption coefficient of the N–H band doubles as the peak shifts from 3360 cm^−1^ to 3320 cm^−1^. They also mentioned that the absorption coefficient changes nonlinearly with the frequency, which means that the same frequency difference does not lead to the same difference in absorption coefficients depending on its frequency location.^27^ The hydrogen bonded N–H in this study is less shifted from the free N–H than the values reported by Skrovanek *et al.*, indicating that the absorption coefficient ratio of the 3335 cm^−1^ peak to the 3383 cm^−1^ peak is expected to be smaller than two but greater than one. Unfortunately, this variable mixture study cannot determine the absorption coefficient ratio independently without knowing the ratio of the two hydrogen-bonded N–H concentrations. When the previously determined *Q* and *R* are used in Fig. 8B, the calculated curves best fit the observed data with the absorption coefficient ratio of 1.6, which is within the expected range from the study by Skrovanek *et al.*^27^

Now, we discuss possible factors that could have affected our spectral analysis and the stoichiometric model for hydrogen bonds in the binary mixture. First, the high-frequency tail of the C=C band may affect the area determination of the hydrogen bonded C=O component at low frequency. As seen in Fig. 1B, both UDMA and TEG-DVBE show the absorption peaks of the C=C stretching mode near the UDMA C=O band at different frequencies with different peak heights. The difference in C=C band shape for different UDMA/TEG-DVBE fractions makes it difficult to subtract the C=C contribution from the C=O peak analysis. However, the majority of C=C contribution, if any, would be originated from UDMA, and the relative contribution of the UDMA C=C band to the total area of the UDMA C=O peak would not change significantly with UDMA mole fraction.

Second, our models assume that all the functional groups are considered separated and behave independently without any geometrical restriction, which is much simpler than the actual molecular picture of the UDMA/TEG-DVBE system. For example, an intramolecular hydrogen bond within a UDMA monomer can occur even at a very low UDMA mole fraction. However, because the N–H spectrum at a low UDMA mole fraction shows a single peak corresponding to the hydrogen bonding to the TEG-DVBE ether oxygen, the intramolecular hydrogen bonding is considered insignificant. Additionally, a hydrogen bond can affect the hydrogen bonding reactivity of its neighbouring functional groups. Proper consideration of this interdependence among functional groups within a molecule will need much more sophisticated models and additional independent measurements of hydrogen bonding in the mixture, which is out of the scope of this study.

Third, the observed large fraction of free C=O at 100% UDMA may be due to the formation of bifurcated hydrogen bonds, where a single acceptor forms hydrogen bonds with two donors.^28,29^ However, the molecules studied for bifurcated hydrogen bonds are either small solvent molecules (*e.g.*, water and methanol) or proteins with prearranged secondary structures. The molecules in this study are much larger than water and methanol, and the bulky side groups near both donors and acceptors in UDMA and TEG-DVBE will sterically hinder the access of two N–H to one C=O. Moreover, the excess number of acceptors in all mixtures makes the probability of bifurcated hydrogen bonding even lower.

Lastly, our assumption of global absorption coefficients and equilibrium constants for each functional group may be oversimplified. For example, the UDMA methacrylate and carbamate C=O groups may have different reactivities. Also, the reactivity of TEG-DVBE ethers next to the styrene group compared to the interior ethers may be different. However, the close center frequencies of the observed subcomponents in the C=O and N–H bands suggest that the effect of location on reactivity is negligible between the same type of functional groups within a molecule.

## Conclusions

We have studied hydrogen bonding interactions in a UDMA/TEG-DVBE mixture by analyzing IR spectra of the C=O and the N–H modes. Simplified stoichiometric models are proposed and tested for the resin monomers, which contain multiple hydrogen bonding donors and acceptors. We have found that at least two competing hydrogen bond acceptor groups from UDMA and one TEG-DVBE acceptor group are involved in hydrogen bonding for UDMA self-association and equally significant inter-association between UDMA and TEG-DVBE. Quantitative information on those monomer interactions helps us to understand the unexpected copolymerization kinetics of this monomer mixture, like the rapid reaction rate and the composition-controlled copolymerization.

## Conflicts of interest

There are no conflicts to declare.

## Disclaimer

Certain commercial equipment, instruments, or materials are identified in this paper in order to adequately specify the experimental procedure. Such identification does not imply recommendation or endorsement by the National Institute of Standards and Technology, nor does it imply that the materials or equipment identified are necessarily the best available for the purpose.

Official contribution of the National Institute of Standards and Technology; not subject to copyright in the United States.

## Supplementary Material

RA-008-C8RA02919A-s001
