# Supplementary material for: Stoichiometric analysis of competing intermolecular hydrogen bonds using infrared spectroscopy
Source: RSC Adv. 2018 Jun 27;8(42):23481–8. doi: 10.1039/c8ra02919a (PMC6205508; doi:10.1039/c8ra02919a)
Supplement: RA-008-C8RA02919A-s001 [file RA-008-C8RA02919A-s001.pdf]

Electronic Supplementary Material (ESI) for RSC Advances.
This journal is © The Royal Society of Chemistry 2018

Electronic Supplementary Information for

Stoichiometric analysis of competing intermolecular hydrogen bonds using infrared spectroscopy

Ian Seungwan Ryu,[†a] Xiaohui Liu,[†b] Ying Jin,[a] Jirun Sun,[*b] Young Jong Lee [*a]

[a] Biosystems and Biomaterials Division, National Institute of Standards and Technology, Gaithersburg, Maryland, 20899, USA
[b] Volpe Research Center, American Dental Association Foundation, Gaithersburg, Maryland, 20899, USA
† These authors contributed equally to this work.
* Corresponding authors: J.S. (jsun@nist.gov) and Y.J.L. (yjlee@nist.gov)

Contents:

1. Derivation of stoichiometric equation (Eq. 8) for (1+1) model
2. Derivation of stoichiometric equation (Eq. 16) for (2+1) model

1. Derivation of stoichiometric equation (Eq. 8) for (1+1) model

$$K_{\mathrm{U}} = \frac{[\mathrm{H}\cdots\mathrm{O_U}]}{[\mathrm{H}][\mathrm{O_U}]} \tag{S1}$$

$$K_{\mathrm{T}} = \frac{[\mathrm{H}\cdots\mathrm{O_T}]}{[\mathrm{H}][\mathrm{O_T}]} \tag{S2}$$

$$P \equiv K_{\mathrm{U}}/K_{\mathrm{T}} \tag{S3}$$

$$[\mathrm{H}\cdots\mathrm{O_U}] + [\mathrm{O_U}] = 4[\mathrm{U}] \tag{S4}$$

$$[\mathrm{H}\cdots\mathrm{O_T}] + [\mathrm{O_T}] = 4[\mathrm{T}] \tag{S5}$$

$$[\mathrm{H}\cdots\mathrm{O_U}] + [\mathrm{H}\cdots\mathrm{O_T}] = 2[\mathrm{U}] \tag{S6}$$

To simplify the equations above, we introduce the fractions of C=O groups satisfying $\Phi + \varphi = 1$.

$$\Phi \equiv \frac{[\mathrm{O_U}]}{[\mathrm{O_U}] + [\mathrm{H}\cdots\mathrm{O_U}]} = \frac{[\mathrm{O_U}]}{4[\mathrm{U}]} \tag{S7}$$

$$\varphi \equiv \frac{[\mathrm{H}\cdots\mathrm{O_U}]}{[\mathrm{O_U}] + [\mathrm{H}\cdots\mathrm{O_U}]} = \frac{[\mathrm{H}\cdots\mathrm{O_U}]}{4[\mathrm{U}]} \tag{S8}$$

Also, the mole fraction of UDMA is defined as described in the main text.

$$\phi_{\mathrm{U}} = \frac{[\mathrm{U}]}{[\mathrm{U}] + [\mathrm{T}]} \tag{S9}$$

Substracting Eq. S6 from S5,

$$[\mathrm{O_T}] = 4[\mathrm{T}] - 2[\mathrm{U}] + [\mathrm{H}\cdots\mathrm{O_U}] \tag{S10}$$

Utilizing Eqs. S7–S10, Eq. S3 can be expressed as following

$$\begin{aligned} P &= \frac{K_{\mathrm{U}}}{K_{\mathrm{T}}} = \frac{[\mathrm{H}\cdots\mathrm{O_U}]}{[\mathrm{O_U}]}\frac{[\mathrm{O_T}]}{[\mathrm{H}\cdots\mathrm{O_T}]} \\ &= \frac{[\mathrm{H}\cdots\mathrm{O_U}]}{[\mathrm{O_U}]}\frac{4[\mathrm{T}] - 2[\mathrm{U}] + [\mathrm{H}\cdots\mathrm{O_U}]}{2[\mathrm{U}] - [\mathrm{H}\cdots\mathrm{O_U}]} \\ &= \frac{1-\Phi}{\Phi}\frac{\frac{4(1-\phi_{\mathrm{U}})}{\phi_{\mathrm{U}}} + 2 - 4\Phi}{-2 + 4\Phi} \end{aligned} \tag{S11}$$

Eq. S11 can be rearranged to the following quadratic equation with respect to Φ, and its solution is Eq. 8 in the main text.

$$2(P-1)\Phi^2 + \left(-P + 1 + \frac{2}{\phi_{\mathrm{U}}}\right)\Phi + \left(1 - \frac{2}{\phi_{\mathrm{U}}}\right) = 0$$

2. Derivation of stoichiometric equation (Eq. 16) for (2+1) model

$$K_{\mathrm{U1}} = \frac{[\mathrm{H}\cdots\mathrm{O}_{\mathrm{U1}}]}{[\mathrm{H}][\mathrm{O}_{\mathrm{U1}}]} \quad (S12)$$

$$K_{\mathrm{U2}} = \frac{[\mathrm{H}\cdots\mathrm{O}_{\mathrm{U2}}]}{[\mathrm{H}][\mathrm{O}_{\mathrm{U2}}]} \quad (S13)$$

$$K_{\mathrm{T}} = \frac{[\mathrm{H}\cdots\mathrm{O}_{\mathrm{T}}]}{[\mathrm{H}][\mathrm{O}_{\mathrm{T}}]} \quad (S14)$$

$$Q \equiv K_{\mathrm{U2}}/K_{\mathrm{U1}} \qquad R \equiv K_{\mathrm{U1}}/K_{\mathrm{T}} \quad (S15)$$

$$[\mathrm{H}\cdots\mathrm{O}_{\mathrm{U1}}] + [\mathrm{O}_{\mathrm{U1}}] = 4[\mathrm{U}] \quad (S16)$$

$$[\mathrm{H}\cdots\mathrm{O}_{\mathrm{U2}}] + [\mathrm{O}_{\mathrm{U2}}] = 4[\mathrm{U}] \quad (S17)$$

$$[\mathrm{H}\cdots\mathrm{O}_{\mathrm{T}}] + [\mathrm{O}_{\mathrm{T}}] = 4[\mathrm{T}] \quad (S18)$$

$$[\mathrm{H}\cdots\mathrm{O}_{\mathrm{U1}}] + [\mathrm{H}\cdots\mathrm{O}_{\mathrm{U2}}] + [\mathrm{H}\cdots\mathrm{O}_{\mathrm{T}}] = 2[\mathrm{U}] \quad (S19)$$

Similar to Eqs. S7 and S8, we introduce the fractions of C=O groups satisfying $\Phi + \varphi = 1$.

$$\Phi \equiv \frac{[\mathrm{O}_{\mathrm{U1}}]}{[\mathrm{O}_{\mathrm{U1}}] + [\mathrm{H}\cdots\mathrm{O}_{\mathrm{U1}}]} = \frac{[\mathrm{O}_{\mathrm{U1}}]}{4[\mathrm{U}]} \qquad \varphi \equiv \frac{[\mathrm{H}\cdots\mathrm{O}_{\mathrm{U1}}]}{[\mathrm{O}_{\mathrm{U1}}] + [\mathrm{H}\cdots\mathrm{O}_{\mathrm{U1}}]} = \frac{[\mathrm{H}\cdots\mathrm{O}_{\mathrm{U}}]}{4[\mathrm{U}]} \quad (S20)$$

From the definition of $Q \equiv K_{\mathrm{U2}}/K_{\mathrm{U1}}$ and Eq. S17 and S20

$$Q\frac{[\mathrm{H}\cdots\mathrm{O}_{\mathrm{U1}}][\mathrm{O}_{\mathrm{U2}}]}{[\mathrm{O}_{\mathrm{U1}}]} + [\mathrm{O}_{\mathrm{U2}}] = 4[\mathrm{U}]$$

$$[\mathrm{O}_{\mathrm{U2}}] = 4[\mathrm{U}]\left(\frac{[\mathrm{O}_{\mathrm{U1}}]}{Q[\mathrm{H}\cdots\mathrm{O}_{\mathrm{U1}}] + [\mathrm{O}_{\mathrm{U1}}]}\right)$$

$$= 4[\mathrm{U}]\left(\frac{\Phi}{Q - Q\Phi + \Phi}\right) \quad (S21)$$

From Eqs. S17–S21,

$$[\mathrm{O}_{\mathrm{T}}] = [\mathrm{H}\cdots\mathrm{O}_{\mathrm{U1}}] - [\mathrm{O}_{\mathrm{U2}}] + 2[\mathrm{U}] + 4[\mathrm{T}]$$

$$= 4[\mathrm{U}](1-\Phi) - 4[\mathrm{U}]\left(\frac{\Phi}{Q - Q\Phi + \Phi}\right) + 2[\mathrm{U}] + 4[\mathrm{T}] \quad (S22)$$

The definition of $R \equiv K_{\mathrm{U1}}/K_{\mathrm{T}}$ can be rearranged into the following Eq. S23

$$R[\mathrm{O}_{\mathrm{U1}}][\mathrm{H}\cdots\mathrm{O}_{\mathrm{T}}] = [\mathrm{H}\cdots\mathrm{O}_{\mathrm{U1}}][\mathrm{O}_{\mathrm{T}}] \quad (S23)$$

Utilizing Eqs. S18, S20–S22 into Eq. S23 and rearranging the resultant Eq. S23 with respect to the mole fraction of UDMA (Eq. S9) lead to Eq. 16 in the main text.

$$\phi_{\mathrm{U}} = \frac{2Q\Phi^2 - 2\Phi^2 - 4Q\Phi + 2\Phi + 2Q}{2(-RQ + R + Q - 1)\Phi^3 + (5RQ - R - 5Q + 1)\Phi^2 + (-3RQ + 4Q + 1)\Phi - Q}$$
